# Identification of the CDH18 gene associated with age-related macular degeneration using weighted gene co-expression network analysis

**DOI:** 10.3389/fgene.2024.1378340

**Published:** 2024-07-16

**Authors:** Guina Liu, Mingqi Tan, Rui Liu, Xuejin Lu, Xiaoshuang Jiang, Yunpeng Bai, Zhigang Guo, Fang Lu

**Affiliations:** ^1^ Department of Ophthalmology, West China Hospital, Sichuan University, Chengdu, China; ^2^ Academy of Medical Engineering and Translational Medicine, Tianjin University, Tianjin, China; ^3^ Department of Cardiac Surgery, Chest Hospital, Tianjin University, Tianjin, China

**Keywords:** age-related macular degeneration, differentially expressed genes, weighted gene co-expression network analysis, hub genes, CDH18

## Abstract

**Purpose:** Age-related macular degeneration (AMD) is a chronic and progressive macular degenerative disease that culminates in a gradual deterioration of central vision. Despite its prevalence, the key biomarkers for AMD have not yet been fully elucidated. In this study, we aimed to efficiently identify biomarkers crucial for diagnosing AMD.

**Methods:** Three datasets pertaining to retinal pigment epithelium (RPE)/choroid tissues associated with AMD were selected from the GEO database. The GSE50195 dataset was utilized to conduct weighted gene co-expression network analysis (WGCNA) for identifying module genes linked to AMD. KEGG and GO enrichment analyses were subsequently conducted on these module genes. GSE29801 and GSE135092 datasets were subjected to differential expression analysis to pinpoint the DEGs intersecting with the module genes. Subsequently, wet AMD (wAMD) and dry AMD (dAMD) mouse models were developed, from which RPE/choroid tissues were harvested to validate the hub genes via RT-qPCR and Western blot.

**Results:** Using the WGCNA, we selected the “antiquewhite4” module (*r* = 0.91 and *p* = 7e-07), which contains a total of 325 genes. Through the intersection of module genes with DEGs, nine hub genes were identified. Pathways involved in complement and coagulation cascades, ECM–receptor interactions, unsaturated fatty acid biosynthesis, and fatty acid elongation play important roles in AMD. Notably, CDH18 demonstrated notable variance across all three datasets. Post validation using RT-qPCR experiments revealed a significant downregulation of CDH18 in both dAMD and wAMD. EGLN3 was expressed at low levels in wAMD. In dAMD, EYA2, LTB, and PODXL were significantly downregulated, whereas APOC1 was notably upregulated. Western blot confirmed that CDH18 was lowly expressed in dAMD and wAMD mouse models.

**Conclusion:** CDH18 was identified as the key gene involved in the pathogenesis of AMD. An imbalance of the complement and coagulation cascades is a potential mechanism of AMD. This study provides a novel idea for diagnosing and treating AMD in the future.

## Introduction

Age-related macular degeneration (AMD) is a chronic and progressive macular degenerative disease leading to the gradual loss of central visual acuity, which is the main cause of blindness worldwide for people over 55 years, following cataracts and glaucoma (Guymer and Campbell, 2023). With global aging, the number of AMD patients will increase accordingly in the coming decades, and it is predicted that the number of global patients with AMD will increase to 288 million by the end of 2040 (Wong et al., 2014). The pathogenetic features of AMD include an increased number and diameter of extracellular retina deposits (drusen), pigmentary irregularity, and progressive atrophy of the retinal pigment epithelium (RPE) and retina (Newman et al., 2012; Somasundaran et al., 2020). However, current therapeutic options for AMD remain limited, partly due to the still unclear understanding of its pathogenetic mechanisms (Fleckenstein et al., 2021).

Previous studies have established that AMD is linked to a variety of risk factors, including advanced age, genetic factors, environmental factors such as smoking, and possibly promising molecular risk factors such as high‐density lipoprotein cholesterol (Heesterbeek et al., 2020; Pugazhendhi et al., 2021). Recent advancements in gene detection methodologies and bioinformatics have led to the identification of more than 50 genetic susceptibility loci for AMD, significantly contributing to its early detection and prognostic prediction (Shughoury et al., 2022). Among all genetic variants, the most common correlated factors are rs10922109 and rs570618 in CFH, rs116503776 in C2/CFB/SKIV2L, rs3750846 in ARMS2/HTRA1, and rs2230199 in C3 (Heesterbeek et al., 2020). However, other genes potentially critical to AMD development have received less attention or are yet to be discovered. For example, a study on bevacizumab treatment for AMD identified an association between the A allele and the homozygous AA genotype of interleukin 8–251A/T and a lack of anatomical response (Hautamäki et al., 2013). Such an in-depth analysis could aid in the development of precise therapies for AMD. Furthermore, it is essential to thoroughly investigate potential genes linked to AMD to improve its diagnosis, treatment efficacy, and prognostic accuracy.

Advancements in the gene chip technology have facilitated the analysis of mRNA-level variations across diverse samples (Buccitelli and Selbach, 2020), facilitating the identification of novel and pivotal genes involved in the pathogenesis of AMD. Public databases, such as the Gene Expression Omnibus (GEO), compile extensive genetic data on various diseases from research institutions globally (Byrd et al., 2020). Systematic network analyses based on gene expression profiles can assist in identifying key genes and disease-related pathways. Traditional analyses of AMD have focused primarily on differentially expressed genes (DEGs), with limited knowledge regarding their co-expression patterns (Li et al., 2022; Liang et al., 2022; Wang et al., 2022). Weighted gene co-expression network analysis (WGCNA) has been widely used to explore key modules and hub genes for the identification of candidate biomarkers and therapeutic targets in diseases like cancers and neuropsychiatric disorders (Li et al., 2018; Jo et al., 2023). Only few studies used the WGCNA to uncover the co-expression network characteristics in AMD (Liang et al., 2022; Han and He, 2023).

In this study, data on AMD from the GEO database were used to identify DEGs, and the WGCNA was conducted at the same time to find the intersection of potential genes. Gene Ontology (GO) enrichment and Kyoto Encyclopedia of Genes and Genomes (KEGG) pathway analyses were further used to reveal possible functions of key modules. Additionally, Gene Set Enrichment Analysis (GSEA) was applied to explore potential biological functions of DEGs. Finally, it is aimed at efficiently identifying genetic biomarkers linked to AMD and providing a theoretical basis for better acknowledgment of the mechanisms of AMD, which would improve the early clinical diagnosis, precise treatment, and prognosis prediction of AMD in the future.

## Materials and methods

**FIGURE 1:**
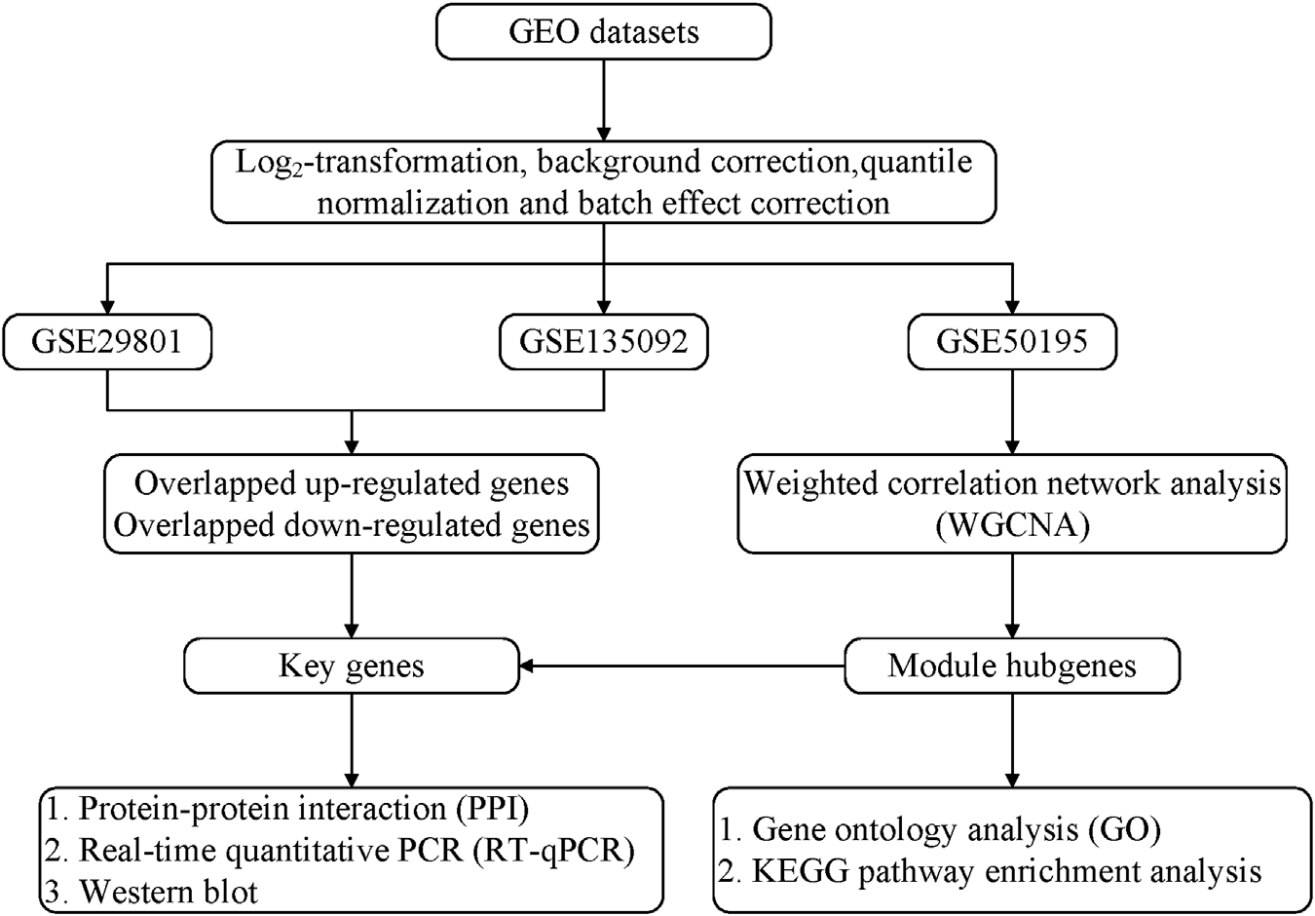
Flowchart of preparation, processing, and analysis.

We present the following article in accordance with the STREGA reporting checklist (Supplementary Table S1). Figure 1 shows the overall workflow of this study.

### Data collection

All microarray datasets were downloaded from the GEO database. We searched the GEO database for microarray datasets using the keyword “age-related macular degeneration.” Datasets were selected if they met the following criteria: 1) were from humans; 2) included expression data from RPE/choroid tissues of both AMD and control macular samples; 3) the number of rows in each platform was >20,000; 4) the number of AMD samples was ≥3, and the number of control samples was ≥3; and 5) there were no repeated samples among the datasets. Finally, three datasets (GSE29801, GSE135092, and GSE50195) were included. Detailed information for these datasets, including the GEO accession ID, dataset country, sample numbers, platform ID, and number of genes in each platform, as well as usage in the current study and references, was recorded and is shown in Supplementary Table S2.

The GSE29801 dataset was generated based on the GPL4133 platform (Agilent-014850 Whole Human Genome Microarray 4 × 44K G4112F), which includes 26 AMD samples and 31 control samples. The GSE135092 dataset was generated by GPL16791 (Illumina HiSeq 2,500 (*Homo sapiens*)) and included 23 AMD samples and 99 control samples. The GSE50195 dataset, based on the GPL17629 platform, included nine AMD samples and eight control samples.

### Data preprocessing

We performed log2 transformation, background correction, and quantile normalization on the expression profiles of GSE50195 using the RMA method from the R package “affy” (Gautier et al., 2004) in R 4.2.1 software (R Foundation for Statistical Computing, Vienna, Austria). We performed log2 transformation and normalization on the expression profiles of GSE29801 and GSE135092 using the voom method from the R package “limma” (Ritchie et al., 2015). Batch normalization of the merged file was conducted using the ComBat method from the R package “sva” (Leek et al., 2012). The probe IDs were converted into gene symbols according to the annotation file. Genes with less than 10 counts of expression in more than one-quarter of the samples were eliminated.

### Differentially expressed genes

Two datasets (GSE29801 and GSE135092) were used to identify DEGs. Differential expression was determined using the limma package in R. For GSE29801, the cutoff values were set as |fold change (FC)| >1.2 and *p* < 0.05. Genes with FC > 1.2 and *p* < 0.05 were highly expressed in AMD, whereas genes with FC < −1.2 and *p* < 0.05 were expressed at low levels in AMD. For GSE135092, genes with FC > 1.5 and *p* < 0.05 were considered to be DEGs. Then, downregulated genes and upregulated genes were separately identified by the intersection of DEGs from these two datasets. Furthermore, the DEGs were visualized as a volcano plot using the “ggplot2” package.

### Weighted gene co-expression network analysis

We selected the top 60% most variant genes in GSE50195 to construct a co-expression network using the “WGCNA” (Langfelder and Horvath, 2008) package. The R package “WGCNA” was used to conduct this analysis and to identify clinical trait-related modules and hub genes. To transform the adjacency matrix to a topological overlap matrix, a soft-threshold power with a scale-free *R* ^2^ near 0.85 and a slope near 1 was selected. We set the soft threshold power to 8 (scale-free *R* ^2^ = 0.84 and slope = −2.69), the cut height to 0.40, and the minimal module size to 30 for network construction and module detection. The module with the highest correlation with AMD was considered the key module. Subsequently, we selected the overlapping DEGs and module genes from the WGCNA as the hub genes of AMD.

### Functional enrichment analysis

KOBAS 3.0 (Bu et al., 2021) was used to test the statistical enrichment of module genes in the Kyoto Encyclopedia of Genes and Genomes (KEGG) and Gene Ontology (GO) pathway enrichment analyses. A *p*-value < 0.05 was considered to indicate statistical significance. The GO and KEGG results were visualized by the R package “ggplot2.”

### Gene set enrichment analysis

The R package “clusterProfiler” (Yu et al., 2012) was used to explore the possible biological functions of the hub genes. To explore the biological signaling pathways involved, gene set enrichment analysis was performed on the DEGs of the GSE29801 and GSE135092 datasets. The database “c2. cp.kegg.v2022.1. Hs.symbols.gmt” was chosen for enrichment. Terms with *p* < 0.05 and FDR<0.25 were considered significant. The GSEA results were visualized by the R packages “GseaVis” and “ggplot2.”

### Protein–protein interaction analysis

After overlapping the DEGs and module genes from the WGCNA, we inputted these genes into the GeneMANIA database (http://genemania.org) to collect information on the interactions of target proteins (Franz et al., 2018).

## Animals

C57BL/6J male mice (6–8 weeks old) were purchased from GemPharmatech Co., Ltd. (Chengdu, China). The animal experiments were all performed according to the ARRIVE guidelines and the ARVO Statement for the Use of Animals in Ophthalmic and Vision. All animal experiments were approved by the Ethical Committee of the West China Hospital, Sichuan University. All animals were given free access to food and drinking water. Mice were housed in a pathogen-free room at constant temperature (22°C) under a 12 h light–dark cycle. Unless otherwise stated, mice were anesthetized with intraperitoneal ketamine (80 mg/kg) and xylazine (12 mg/kg) in this study. The pupils were dilated with an eye drop containing 0.5% tropicamide and 0.5% phenylephrine hydrochloride. The dry AMD (dAMD) mouse model was induced by NaIO_3_. The wet AMD (wAMD) mouse model was induced by a laser. The control group did not receive any treatment.

### NaIO_3_-induced dAMD

NaIO_3_ (Macklin, Shanghai, China) was dissolved in sterile saline and intraperitoneally injected into mice at a concentration of 40 mg/kg.

### Laser-induced wAMD

Lateral induction of choroidal neovascularization (CNV) in mice was performed by an image-guided laser system (Micron IV, Phoenix Research Laboratories) in accordance with the method described by Gong et al. (2015). After anesthesia and pupil dilation, 2.5% hypromellose was applied to the mouse cornea. The laser settings were as follows: wavelength, 532 nm; diameter, 50 μm; duration, 70 m; and power, 260 mW. Three or four laser burns were induced around the optic disc. The distance between the two laser burns and between the laser burn and the optic disc was approximately double the diameter of the optic disc. The success of the operation was confirmed by the formation of a bubble and haze area around the lesion immediately after laser photocoagulation.

### mRNA isolation and real-time qPCR

On day 7 post-NaIO_3_ or laser treatment, total RNA from RPE/choroid tissues was extracted using an RNAprep Pure Blood Kit (RC101, Vazyme, Nanjing, China) following the manufacturer’s protocol. Quantitative gene expression analysis via reverse transcription-quantitative PCR was performed to validate hub gene expression. Total RNA extracted from RPE/choroid tissues was reverse transcribed into cDNA using a Hifair^®^ III 1st Strand cDNA Synthesis SuperMix kit for qPCR (gDNA digester plus) (11141ES, YEASEN, Shanghai, China). The following reaction conditions were used: 42°C for 2 min, 25°C for 5 min, 55°C for 15 min, and 85°C for 5 min.

The RT-qPCR experiments were performed using a Hieff UNICON^®^ universal Blue qPCR Master Mix kit (11184ES, YEASEN, Shanghai, China) in a 7,500 Fast Real-Time PCR system (Applied Biosystems, San Francisco, CA, United States). The following thermocycling conditions were used: 95°C for 2 min, followed by 40 cycles at 95°C for 10 s, 60°C for 30 s, 95°C for 15 s, 60°C for 60 s, 95°C for 15 s, and 60°C for 15 s for the final extension. Standard and melting curves were generated for every plate for each gene to ensure that the reaction was efficient and specific. The cycle threshold value of β-actin served as the internal control. The relative expression levels of different genes were analyzed via the 2^−ΔΔCT^ method.

Primer sequences were obtained from PrimerBank (http://pga.mgh.harvard.edu/primerbank) and synthesized by Sangon Biotechnology (Shanghai, China) (Supplementary Table S1 **)**.

### Protein extracting and Western blotting

Protein extracts were obtained from the RPE–choroid complexes of mice at 7 days after laser injury or injecting NaIO_3_. The concentration of protein was evaluated using BCA Protein Assay Kits (Thermo Scientific, catalog no. 23227). Equivalent amounts of protein were electrophoresed on 10% SDS–polyacrylamide gels. The protein was electrotransferred to a polyvinylidene difluoride membrane (Merck Millipore), which was then blocked in a solution of 5% skim milk powder in tris-buffered saline with Tween 20 (TBST, pH 7.5) for 2 h at room temperature and probed overnight at 4°C with primary antibodies against CDH18 (1:1,000; Invitrogen, catalog no. PA5-36240) or glyceraldehyde-3-phosphate dehydrogenase (GAPDH) (1:1,000; Proteintech, catalog no. 60004-1-Ig). After the membranes were washed with TBST, horseradish peroxidase-conjugated secondary antibodies were applied (1:10,000; ZSGB-BIO, catalog no. ZB2301 and ZB2305) for 1 h at room temperature. The signals were visualized and recorded using an enhanced chemiluminescence kit (Merck Millipore) and a molecular imaging system (Amersham Imager 600, GE Healthcare). Protein amounts were quantified by densitometry and normalized to amounts of GAPDH. The experiment was replicated three times using independent biological samples.

### Statistical analysis

Statistical analysis was performed with R 4.2.1 software (R Foundation for Statistical Computing, Vienna, Austria) and GraphPad Prism 9.0 software (GraphPad Software, Inc., La Jolla, CA) using an independent sample *t*-test when comparing the two groups. *P* < 0.05 was considered statistically significant.

## Results

### DEGs in the RPE between AMD and normal samples

**FIGURE 2:**
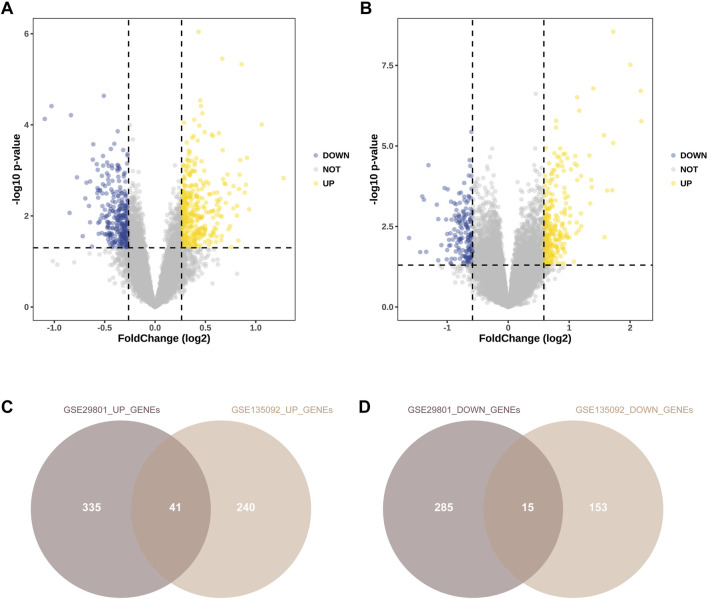
Identification of DEGs of RPE between AMD and normal samples. Volcano plots of differential gene expression data from GSE29801 **(A)** and GSE135092 **(B)**. In the volcano plots, the red points show upregulated genes (fold change (FC) > 1.2 and *p* < 0.05 in GSE29801; fold change (FC) > 1.5 and *p* < 0.05 in GSE135092), whereas the blue points represent downregulated genes (fold change (FC) < −1.2 and *p* < 0.05 in GSE29801; fold change (FC) < −1.5 and *p* < 0.05 in GSE135092). **(C)** The overlapped upregulated genes from GSE29801 and GSE135092. **(D)** The overlapped downregulated genes from GSE29801 and GSE135092. DEG, differentially expressed gene; RPE, retinal pigment epithelium; AMD, age-related macular degeneration; FC, fold-change.

After preprocessing of the GSE29801 and GSE135092 datasets, DEGs were identified according to the *p* and |fold-change (FC)| values. In GSE29801, DEGs were identified if the cutoff value met the criteria of a |fold change (FC)| >1.2 and *p* < 0.05. In GSE135092, DEGs were identified if the cutoff value met the criteria of |fold change (FC)| >1.5 and *p* < 0.05. A volcano plot of all the genes is shown in Figure 2. We identified 376 upregulated and 300 downregulated genes in GSE29801 (Figure 2A) and 281 upregulated and 168 downregulated genes in GSE135092 (Figure 2B). Additionally, we removed the duplicated genes among the upregulated and downregulated genes from these two datasets. Finally, 41 upregulated DEGs and 15 downregulated DEGs were identified in both datasets (Figures 2C,D).

### WGCNA and key module identification

**FIGURE 3:**
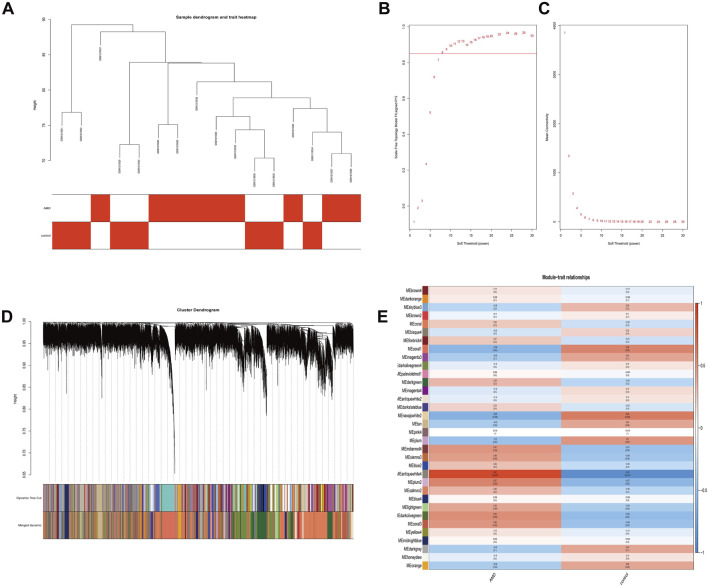
Sample clustering, network construction of WGCNA, and key module identification. **(A)** Clustering dendrogram of nine AMD samples and seven control samples. The color intensity was proportional to disease status (AMD or normal samples). **(B)** The scale-free fit index. **(C)** Mean connectivity for various soft-thresholding powers. The soft-thresholding power of 8 was selected based on the scale-free topology criterion. **(D)** Network heatmap plot of all genes. **(E)** Heatmap of the correlation between module eigengenes and the disease status of AMD. The corresponding correlation coefficient along with the *p*-value is given in each cell, and each cell is color-coded by correlation according to the color (legend at right). The antiquewhite4 module was most significantly correlated with AMD. WGCNA, weighted gene co-expression network analysis; AMD, age-related macular degeneration; RPE, retinal pigment epithelium.

The GSE50195 dataset was used to construct a co-expression network via the WGCNA. The hierarchical clustering tree was created based on the dynamic hybrid cut (Figure 3A), with a scale-free network and topological overlaps. Based on the scale-free topology criterion, a soft-thresholding power of 8 was selected (scale-free *R* ^2^ = 0.84 and slope = −2.69; Figures 3B,C). A heatmap depicted the high co-expression of all genes (Figure 3D). Moreover, a total of 34 modules were identified for further analysis (Figure 3E). Among these 34 modules, we distinguished key modules between the AMD and control samples. Antiquewhite4 had the strongest correlation (r = 0.91 and *p* = 7e-07) with AMD. Therefore, we identified the antiquewhite4 module as the key module, and a total of 325 genes were included in this module for further analysis.

### Enrichment analysis of key modules

**FIGURE 4:**
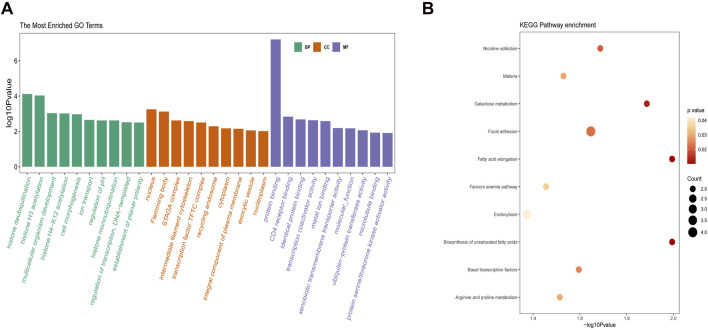
Enrichment analysis of the key module. **(A)** GO analysis on the antiquewhite4 module. **(B)** KEGG pathway on the antiquewhite4 module. The significance of enrichment gradually increases from yellow to red, and the size of the dots indicates the number of genes contained in the corresponding pathway. GO, gene ontology; KEGG, Kyoto Encyclopedia of Genes and Genomes.

Functional enrichment analysis of the antiquewhite4 module was performed based on the GO and KEGG databases. As shown in Figure 4A, the ontology was composed of three domains (biological process, cellular component, and molecular function). The enriched biological processes were mainly involved in histone deubiquitination, histone H3 acetylation, and multicellular organism development. The cellular components were mainly enriched in the nucleus, flemming body, and STAGA complex, whereas the enriched molecular functions were mainly involved in protein binding, CD4 receptor binding, and identical protein binding. The KEGG pathway analysis results are shown in Figure 4B. Fatty acid elongation and biosynthesis of unsaturated fatty acids were the most enriched pathways, followed by galactose metabolism, focal adhesion, and basal transcription factor pathways.

### GSEA of DEGs

**FIGURE 5:**
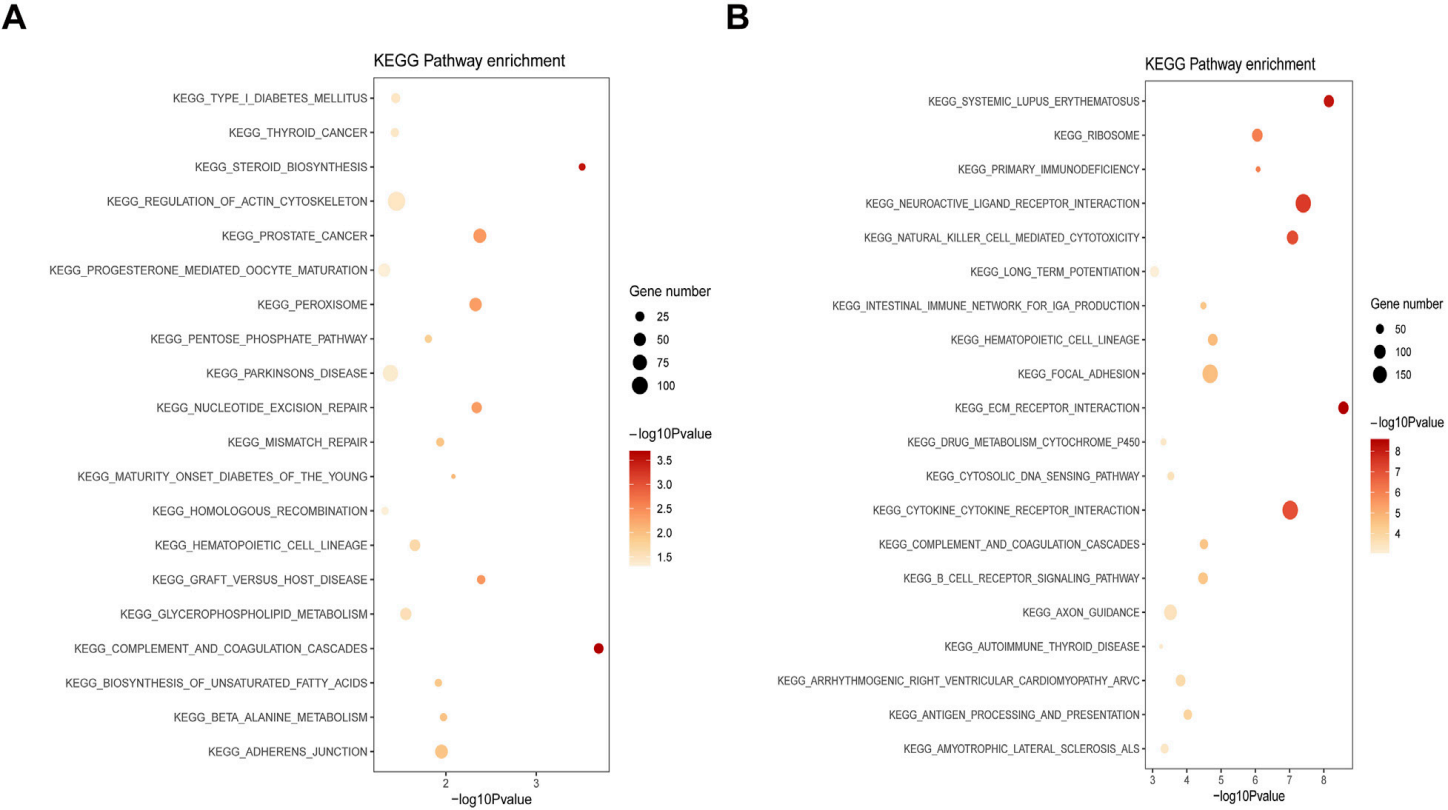
GSEA analysis of DEGs from the GSE29801 and GSE135092 datasets. **(A)** The correlated signaling pathway of DEGs from the GSE29801 dataset. **(B)** The correlated signaling pathway of DEGs from the GSE135092 dataset. The significance of enrichment gradually increases from yellow to red, and the size of the dots indicates the number of genes contained in the corresponding pathway. GSEA, gene set enrichment analysis; DEG, differentially expressed gene.

Based on the DEGs from the GSE29801 and GSE135092 datasets, GSEA was performed to reveal the potential biological functions of the DEGs. For DEGs from the GSE29801 dataset, we found that two main signaling pathways from KEGG were correlated: complement and coagulation cascades and steroid biosynthesis (Figure 5A). Similarly, for DEGs from the GSE135092 dataset, several main signaling pathways correlated from KEGG were extracellular matrix (ECM)–receptor interaction, systemic lupus erythematosus, neuroactive ligand–receptor interaction, natural killer cell-mediated cytotoxicity, and cytokine–cytokine receptor interaction (Figure 5B).

### PPI network construction and identification of hub genes

**FIGURE 6:**
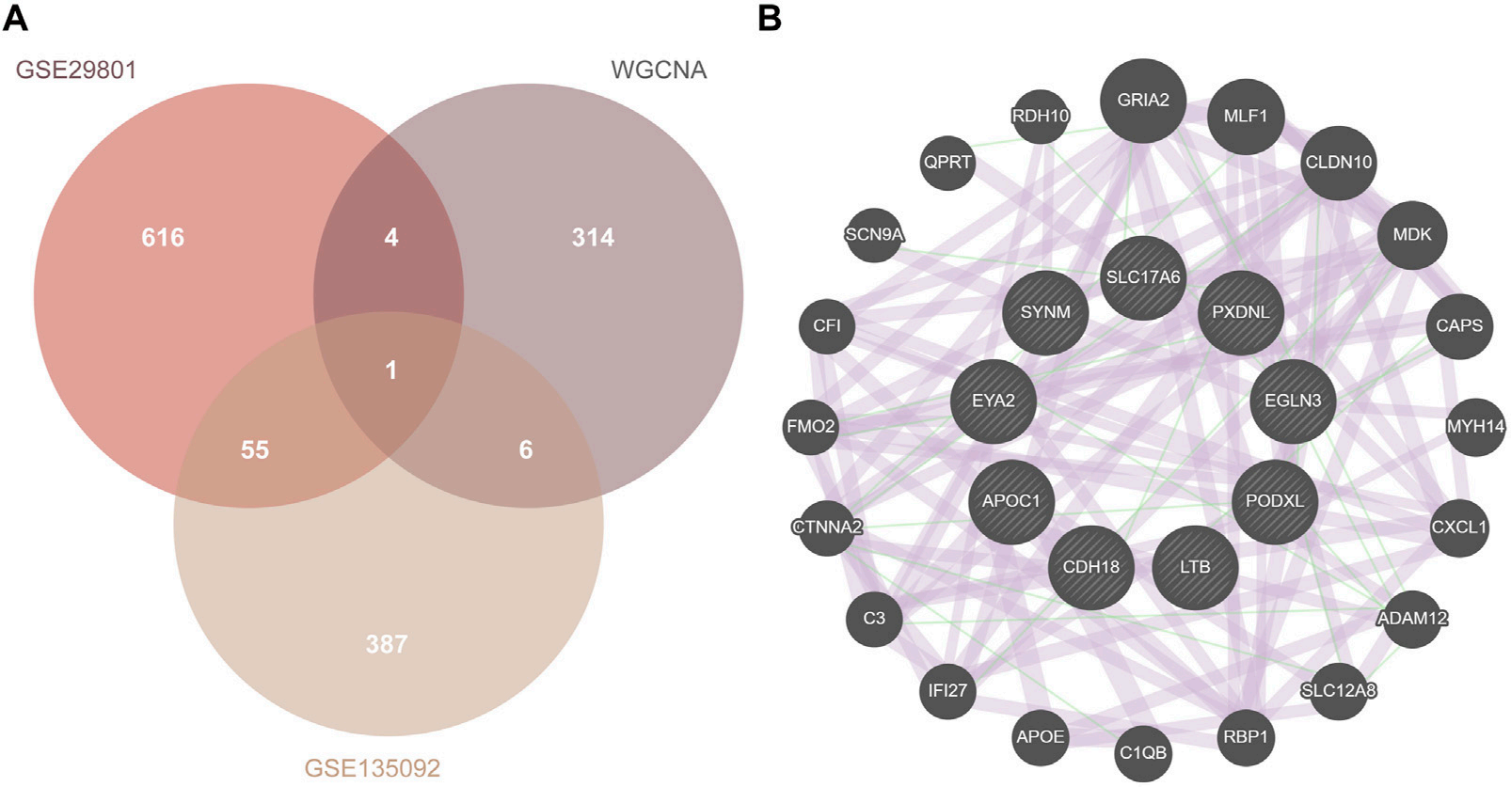
PPI network construction. **(A)** Overlapped genes of DEG union and module genes from WGCNA. **(B)** The PPI network of hub genes using the GeneMANIA database. PPI, protein–protein interaction; DEG, differentially expressed gene; WGCNA, weighted gene co-expression network analysis.

After overlapping the identified DEGs and module genes (325 genes) from the WGCNA, a total of 11 potential genes were screened out (Figure 6A). Five genes (APOC1, SYNM, SLC17A6, PXDNL, and LINC02159) were upregulated, whereas 6 genes (EYA2, PODXL, EGLN3, LTB, CDH18, and LINC00861) were downregulated. LINC02159 and LINC00861 were excluded because of their association with lncRNAs. Finally, 9 hub genes were identified, and 20 co-expressed genes from outer databases were predicted using the GeneMANIA database (Figure 6B). Additionally, the CDH18 gene overlapped with DEGs from GSE29801, DEGs from GSE135092, and module genes from the WGCNA. The 20 co-expressed genes are as follows: GRIA2, MLF1, CLDN10, MDK, CAPS, MYH14, CXCL1, ADAM12, SLC12AB, RBP1, C1QB, APOE, IFI27, C3, CTNNA2, FMO2, CF1, SCN9A, QPRT, and RDH10. The main functions of the hub genes were complement activation, lipoprotein particle formation, cholesterol esterification, synapse organization, and neuro-apoptotic processes.

### Validation of the expression of hub genes

**FIGURE 7:**
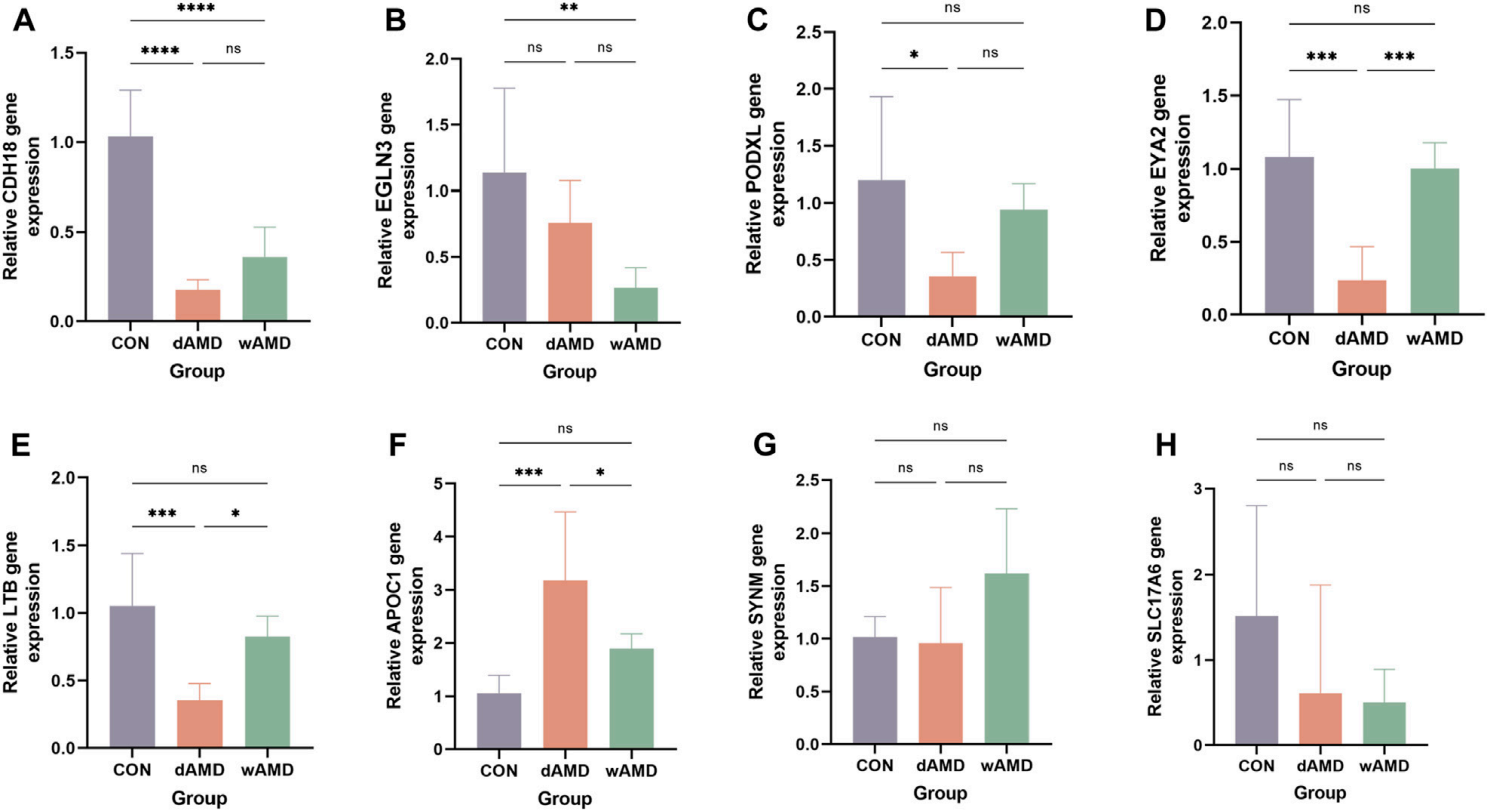
Animal subject validation of gene expression. **(A)** Relative expression of CDH18, decreasing in both dAMD and wAMD, consistent with the result from online datasets. **(B)** Relative mRNA expression value of EGLN3, decreasing in dAMD (*p* < 0.01) and no difference in wAMD (*p* > 0.05). **(C)** Relative mRNA expression value of EYA2, decreasing in dAMD (*p* < 0.001) and no difference in wAMD (*p* > 0.05). **(D)** Relative mRNA expression value of LTB, decreasing in dAMD (*p* < 0.01) and no difference in wAMD (*p* > 0.05). **(E)** Relative mRNA expression value of PODXL, decreasing in dAMD (*p* < 0.05) and no difference in wAMD (*p* > 0.05). **(F)** Relative mRNA expression value of APOC1, increasing in dAMD (*p* < 0.001) and no difference in wAMD (*p* > 0.05). **(G)** Relative mRNA expression value of SYNM, no difference in dAMD and wAMD (*p* > 0.05). **(H)** Relative mRNA expression value of SLC17A6, no difference in dAMD and wAMD (*p* > 0.05). All data were detected by RT-qPCR (n = 6). *p < 0.05, **p < 0.01, ***p < 0.001, and ****p < 0.0001.

**FIGURE 8:**
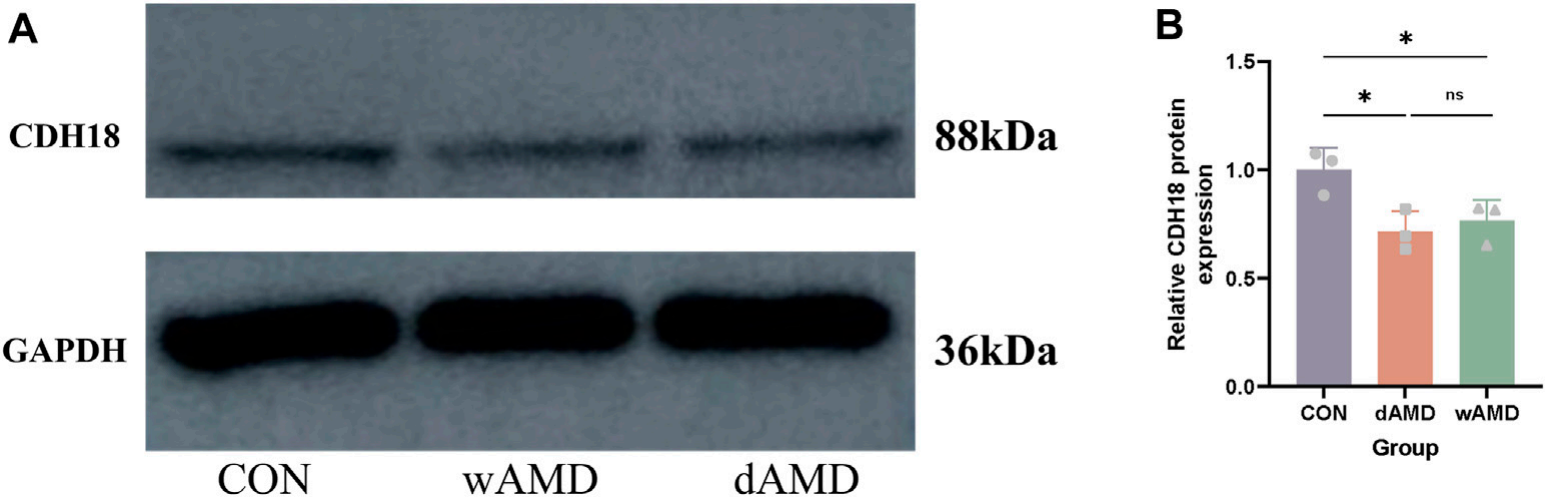
Validation of CDH18 protein. **(A)** Western blotting of RPE–choroid complexes from normal mice and dAMD and wAMD mouse models. **(B)** Relative expression of the CDH18 protein among normal mice and dAMD and wAMD mouse models (n = 3). *p < 0.05.

RT-qPCR experiments were performed to determine the expression of the hub genes, except for PXDNL, which was not detected in the mice. Finally, RT-qPCR of eight genes was performed (Figure 7). The detection of total mRNA from RPE/choroid tissues showed that CDH18 expression in both the dAMD and wAMD model groups was significantly lower than that in the control group (Figure 7A, *p* < 0.0001), which was consistent with the results from public datasets (Supplementary Figure S1). The relative expression of EYA2, LTB, and PODXL in the dAMD group was significantly lower than that in the control group (*p* < 0.05), whereas the expression of EGLN3 was downregulated in the wAMD group (*p* < 0.05, Figure B ∼ E). APOC1 expression was increased in the dAMD group (*p* < 0.001), whereas it only showed an increasing trend in the wAMD group (*p* > 0.05, Figure 7F). However, no difference was found in SYNM between the dAMD and wAMD groups and between the dAMD and wAMD groups and the control group (*p* > 0.05, Figure 7G). SLC17A6 seemed to have decreased expression in both the dAMD and wAMD groups, which was opposite to the results of the analysis of the online datasets (*p* > 0.05, Figure 7H). Additionally, the CDH18 protein was lowly expressed in both dAMD and wAMD mouse models through Western blotting (*p* < 0.05, Figure 8).

## Discussion

In this study, we analyzed the gene expression of genes in the RPE/choroid tissues, aiming to identify hub genes in AMD patients. In contrast to previous research (Li et al., 2022; Liang et al., 2022; Wang et al., 2022), our approach utilized three gene expression profiles comprising 55 AMD samples and 138 control samples. Two of these datasets were used to identify DEGs, whereas the third dataset was used to analyze key gene network modules through the WGCNA. Ultimately, with a primary focus on the modules identified by the WGCNA, our analysis concentrated on exploring the genes within the key module that intersect with the DEGs from the GSE29801 and GSE135092 datasets. The intersection results revealed that CDH18 exhibited significant differences in expression across the three datasets, followed by eight other hub genes that overlapped between the two profiles.

AMD is a complex, multifactorial, progressive disease in which multiple molecular pathways regulate its pathogenesis (Choudhary and Malek, 2019). Early stages of AMD primarily affect the outer retina due to the accumulation of uncleared cellular debris known as drusen. As the disease progresses, it may evolve into dAMD or wAMD (Thomas et al., 2021). dAMD is characterized by a gradual loss of visual function, which is attributed to the deterioration of the choriocapillaris, atrophic loss of the outer retina, and eventual disruption and death of the photoreceptor layer. In contrast, wAMD involves the growth of new blood vessels that disrupt and damage the photoreceptor layer, significantly impairing vision. These abnormal vessels typically originate from the choroidal circulation in wAMD; however, neovascularization and leakage can also stem from the retinal vasculature (Flores et al., 2021; Guymer and Campbell, 2023). Given the challenges in obtaining living tissue from patients for research, mouse models are commonly employed to study both dAMD and wAMD. In this study, NaIO3 and laser treatments were used to induce dAMD and wAMD in mouse models, respectively. These are classical models for studying AMD. The dAMD model primarily represents the atrophy of RPE (Chowers et al., 2017), whereas the wAMD model predominantly reflects choroidal neovascularization (van Lookeren Campagne et al., 2014). For this research, the RPE/choroid complex was utilized to validate, to some extent, the gene expression of RPE/choroid tissues from patients using public datasets.

CDH18 encodes a type II classical cadherin from the cadherin superfamily of integral membrane proteins that mediate calcium-dependent cell‒cell adhesion, playing important roles in neurogenesis, neuron migration, and axon growth (Lin et al., 2014). Various studies have demonstrated that genetic abnormalities in CDH18 are associated with various neuropsychiatric disorders, such as bipolar disorder (Chen et al., 2017), schizophrenia (Redies et al., 2012), depression (Chen et al., 2017), and autism (Redies et al., 2012), as well as cancers, such as colorectal cancer (Venkatachalam et al., 2011) and ovarian cancer (Chmelarova et al., 2019). Currently, there is no direct evidence to establish a clear association between CDH18 and AMD. However, inferring from previous research findings, a potential relationship between the two may exist. A study by Zhao et al. (2023) reported that the downregulation of CDH18 in gastric cancer cell lines may lead to the activation of the PI3K/AKT signaling pathway. Simultaneously, Sun et al. (2023) reported that the activation of the PI3K/AKT signaling pathway was associated with RPE dysfunction and choroidal neovascularization in AMD patients. Therefore, we hypothesize that CDH18 might be implicated in AMD development via its regulatory role in the PI3K/AKT signaling pathway. Additionally, prior research on the epicardium revealed that the loss of CDH18 is associated with the onset of epicardial-to-mesenchymal transition (EMT) (Junghof et al., 2022). Given this theory and considering the contribution of EMT to the AMD pathology (Shu et al., 2020; Li et al., 2023), we hypothesized that the abnormal expression of CDH18 could lead to AMD via EMT.

PODXL encodes transmembrane antiadhesive sialomucin, which is ubiquitously expressed on the luminal surface of endothelial cells in capillaries (Siemerink et al., 2016). PODXL is highly expressed in the adult retina (Lakowski et al., 2011), but its expression was decreased in a dAMD mouse model. This decrease in expression may be linked to the atrophy of cone photoreceptors, as Michael et al. (Kaufman et al. (2019) identified PODXL as an early marker of developing cone photoreceptors and suggested that its anti-adhesive role may prevent the clumping of cones. EGLN3 enables peptidyl-proline 4-dioxygenase activity and responds to hypoxia by targeting hypoxia-inducible factor-1α (HIF-1α) for degradation (Forooghian et al., 2007). Reduced expression of EGLN3 can lead to the accumulation of HIF‐1α, resulting in increased endothelial growth factor receptor levels in wAMD (Babapoor-Farrokhran et al., 2023; Zhang et al., 2023). However, no significant differences in PODXL or EGLN3 expression were detected between the wAMD and dAMD groups, suggesting that these two genes may not be suitable biomarkers for distinguishing the subtypes of AMD.

APOC1 encodes a member of the apolipoprotein C1 family and is associated with monocyte differentiation and lipoprotein metabolism. APOC1 is highly expressed in younger patients with dementia (Tysoe et al., 1998) and diabetic nephropathy (Hirano et al., 2003), and in the aqueous humor of diabetic retinopathy patients with type 2 diabetes (Saucedo et al., 2022). EYA2 encodes a member of the eyes absent family of proteins that are expressed relatively late in visual system formation (Duncan et al., 1997) and has been shown to regulate the formation of retinal ganglion cells (Gao et al., 2014). LTB is a type II membrane protein of the tumor necrosis factor family that induces the inflammatory response system and is involved in the normal development of lymphoid tissue (Remouchamps et al., 2011). Genetic deficiency in the LTB leads to disrupted splenic architecture, the absence of lymphoid follicles, and the absence of follicular dendritic cell networks (Tani et al., 2004), affecting the balance between autoimmune and infectious pathologies (Spahn et al., 2006). However, no research has focused on the expression of the above three genes in AMD, and the underlying mechanisms remain unclear. Our results first indicated that APOC1 was highly expressed in dAMD mice, whereas EYA2 and LTB were expressed at low levels in these models. Additionally, differences in the expression of APOC1, EYA2, and LTB between dAMD and wAMD suggested that these genes may be potential biomarkers for distinguishing these two forms of AMD.

Enrichment analysis indicated that DEGs and the key module interacted in AMD disease through complement and coagulation cascades, ECM–receptor interactions, unsaturated fatty acid biosynthesis, and fatty acid elongation pathways. Proteomic profiling of the humor revealed that complement components were associated with macular degeneration and pathological processes, including choroidal neovascularization, geographic atrophy, and retinal drusen (Rinsky et al., 2021; Santos et al., 2023). Furthermore, proteomics analysis of peripheral blood and urine showed that complement and coagulation cascades were dysregulated in AMD samples and that complement factors H and C3 could help in the differentiation of the subtypes of AMD (Sivagurunathan et al., 2021). Another significant aspect of the AMD pathology is attributed to disorders in the regulation of the ECM (Nita et al., 2014), including multiple proteins, such as metalloproteinases (Marin-Castaño et al., 2003; Pons et al., 2011), integrins (Bhatwadekar et al., 2020; Mrugacz et al., 2021), and complement factors (Fernandez-Godino and Pierce, 2018). Long-chain polyunsaturated fatty acids (LC-PUFAs) have been linked to AMD pathogenesis through epidemiologic, biochemical, and genetic studies (Skowronska-Krawczyk and Chao, 2019). Clinical trial results indicated that a high dietary intake of (n-3) LC-PUFAs was associated with a low risk of developing AMD (Sangiovanni et al., 2009; Christen et al., 2011; Merle et al., 2013). Specifically, individuals consuming foods rich in (n-3) LC-PUFAs had a 30% lower risk of developing central geographic atrophy and were 50% less likely to develop AMD than those with the lowest intake (Sangiovanni et al., 2009).

There are several limitations in this study. First, our research did not include all the available datasets; instead, we focused on three specific datasets that had been previously overlooked in the literature to reveal novel hub genes in AMD. Second, our focus was exclusively on gene expression in the RPE/choroid tissues of AMD patients from the public datasets, which represents only a part of the complex pathogenesis of AMD, because the clinical change in AMD emerges not only in RPE/choroid tissues but also in retina tissues and even the whole eyeball. Third, due to the absence of the PXDNL gene in mice, its expression could not be validated through RT-qPCR in mouse models, so the later research would focus on the human cell lines to reveal the potential function of the PXDNL gene. Fourth, the difficulty in obtaining RPE/choroid tissues from humans may reduce the potential application of CDH18 and other hub genes in the clinical practice. Finally, the classical mouse models of NaIO3-induced dAMD and laser-induced wAMD only partially replicate the features of AMD, as observed in humans, mainly for the mice’s leaky macula area, which is the main site of the AMD pathological change.

In conclusion, bioinformatics analysis provided evidence for investigating potential biomarkers of AMD. We identified CDH18 as a potential key factor involved in AMD and first validated the expression of five other hub genes (PODXL, EGLN3, APOC1, EYA2, and LTB) in AMD. Moreover, complement and coagulation cascades, ECM–receptor interactions, unsaturated fatty acid biosynthesis, and fatty acid elongation were found to play important roles in AMD. This study may provide a novel approach for diagnosing AMD and revealing the potential mechanism of AMD.

## Data Availability

The original contributions presented in the study are included in the article/Supplementary Material; further inquiries can be directed to the corresponding author.
