# Supplementary material for: Identification of the CDH18 gene associated with age-related macular degeneration using weighted gene co-expression network analysis
Source: Front Genet. 2024 Jul 16;15:1378340. doi: 10.3389/fgene.2024.1378340 (PMC11286549; doi:10.3389/fgene.2024.1378340)
Supplement: Supplementary file 1 [file Table1.DOCX]

**supplementary table 2 detailed information of included datasets.**

| GEO ID | country | sample numbers | platform | number of probe in platform | references |
| --- | --- | --- | --- | --- | --- |
| GSE29801 | USA | 31normal/26AMD | GPL4133 | 45220 | doi: 10.1186/gm315. |
| GSE135092 | USA | 99normal/23AMD | GPL16791 | 58302 | doi:10.1016/j.celrep.2019.12.082. doi:10.3389/fcell.2023.1252547. |
| GSE50195 | USA | 8normal/9AMD | GPL17629 | 42187 | PMID: 24265543 |
