# Supplementary figures and images for: Identification of the CDH18 gene associated with age-related macular degeneration using weighted gene co-expression network analysis
Source: Front Genet. 2024 Jul 16;15:1378340. doi: 10.3389/fgene.2024.1378340 (PMC11286549; doi:10.3389/fgene.2024.1378340)

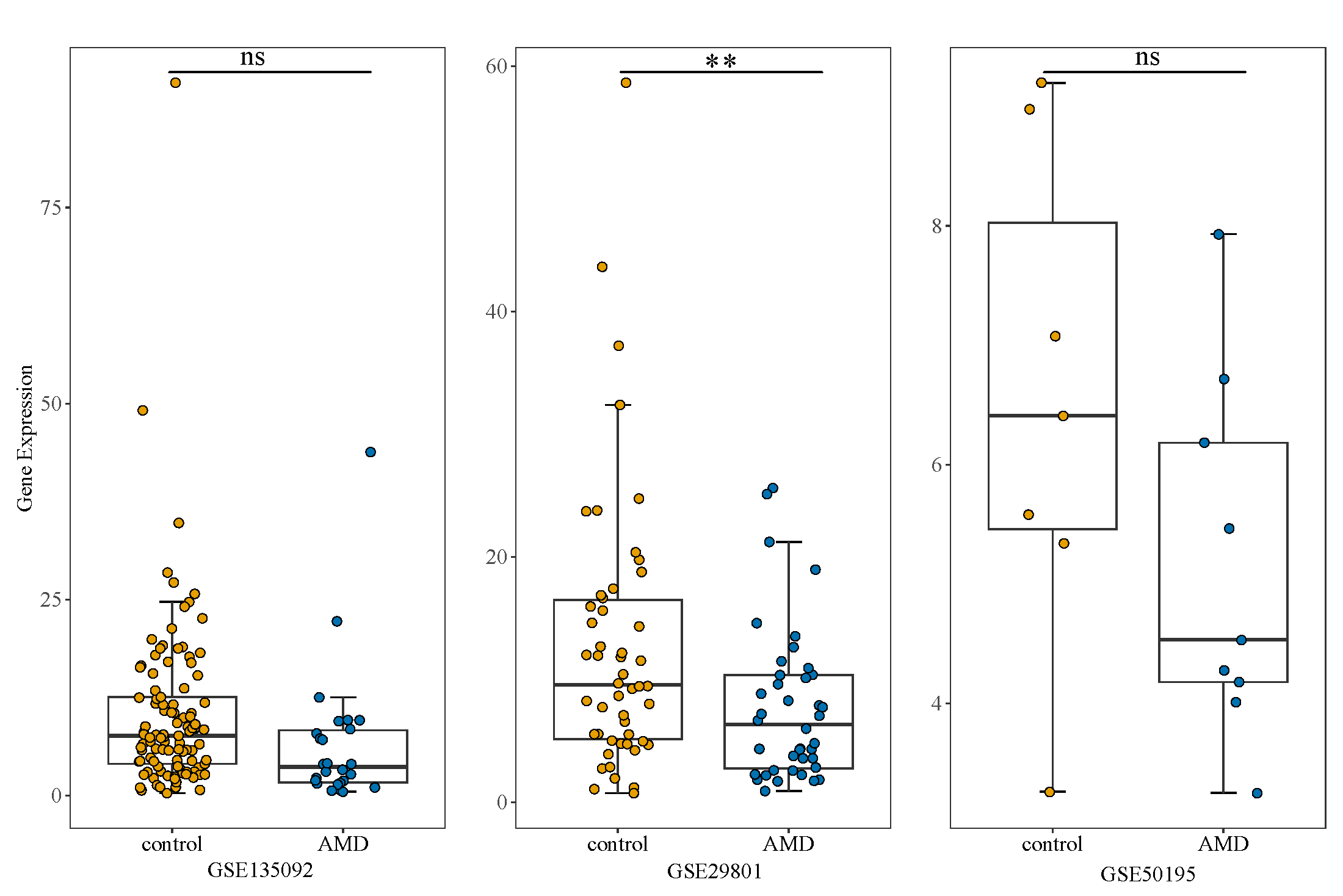

Supplement: Supplementary file 2 [file Image1.TIF]
